# Evaluating ChatGPT as an adjunct for the multidisciplinary tumor board decision-making in primary breast cancer cases

**DOI:** 10.1007/s00404-023-07130-5

**Published:** 2023-07-17

**Authors:** Stefan Lukac, Davut Dayan, Visnja Fink, Elena Leinert, Andreas Hartkopf, Kristina Veselinovic, Wolfgang Janni, Brigitte Rack, Kerstin Pfister, Benedikt Heitmeir, Florian Ebner

**Affiliations:** 1 Department of Gynecology and Obstetrics, University Hospital Ulm, Prittwitzstr. 43, 89075 Ulm, Germany; 2 Gynäkologische Gemeinschaftspraxis Freising & Moosburg, Munich, Germany

**Keywords:** Artificial intelligence, Breast cancer, Multidisciplinary tumor board, ChatGPT

## Abstract

**Background:**

As the available information about breast cancer is growing every day, the decision-making process for the therapy is getting more complex. ChatGPT as a transformer-based language model possesses the ability to write scientific articles and pass medical exams. But is it able to support the multidisciplinary tumor board (MDT) in the planning of the therapy of patients with breast cancer?

**Material and Methods:**

We performed a pilot study on 10 consecutive cases of breast cancer patients discussed in MDT at our department in January 2023. Included were patients with a primary diagnosis of early breast cancer. The recommendation of MDT was compared with the recommendation of the ChatGPT for particular patients and the clinical score of the agreement was calculated.

**Results:**

Results showed that ChatGPT provided mostly general answers regarding chemotherapy, breast surgery, radiation therapy, chemotherapy, and antibody therapy. It was able to identify risk factors for hereditary breast cancer and point out the elderly patient indicated for chemotherapy to evaluate the cost/benefit effect. ChatGPT wrongly identified the patient with Her2 1 + and 2 + (FISH negative) as in need of therapy with an antibody and called endocrine therapy “hormonal treatment”.

**Conclusions:**

Support of artificial intelligence by finding individualized and personalized therapy for our patients in the time of rapidly expanding amount of information is looking for the ways in the clinical routine. ChatGPT has the potential to find its spot in clinical medicine, but the current version is not able to provide specific recommendations for the therapy of patients with primary breast cancer.

## What does this study add to the clinical work

**Table:** 

Our study provides information about usability of ChatGPT in the multidisciplinary tumor boards as supportive tool for decision making process in breast cancer patients. So far as we know, it is the first study regarding this topic.	

## Introduction

The daily rising number of studies providing new data about breast cancer (BC) is huge. Advances in the therapy of BC lead to more personalized and individualized treatment, and the therapeutic decision-making process is getting more and more complex. So, the establishment of a multidisciplinary tumor board (MDT) was the first step to optimizing patient care. It is a cost-effective tool and improves patients´ survival [1]. Nevertheless, on February 28th, 2023, there were more than 6000 publications regarding BC from the year 2023 available in PubMed suggesting approximately 3000 papers per month. It is barely possible for a human being to concentrate all available information and connect them with the particular patient in order to provide highly individualized and personalized therapeutic options.

Moreover, there are already various programs developed to support the therapeutic planning and predict a risk of disease [2–4]. These are developed on the base of human-programmed algorithms in order to identify the best possible therapy for the particular patient and some of them use an artificial intelligence (AI) with the ability of machine learning (ML) [3, 4]. AI already accompanies the area of breast cancer, especially in radiology in so-called computer-assisted diagnosis in mammography screening [5] or in radiation oncology [6]. There are data about using Watson IBM in the planning of the treatment of the patients with BC showing promising results [4].

One of the youngest development of AI based chatbots is ChatGPT (OpenAI, San Francisco) what is due to some opinions considered as one of the best on the market [7, 8]. It possesses the ability to write high-quality scientific papers [8]. That implies the ability to work with information and databases and due to the developer, it is able “to answer follow-up questions, admit its mistakes, challenge incorrect premises, and reject inappropriate requests” [9, 10]. Additionally, the ChatGPT can pass the United States Medical Licensing Exam [10]. Considering all mentioned abilities, the signs of critical thinking as evaluating, analyzing, synthesizing, can be recognized and thanks to its development based on Reinforcement Learning from Human Feedback ChatGPT could be an enrichment of current MDT.

But can the best available AI enhance the ability of a MDT-member by making a correct decision in the therapy planning? The goal of our study is to evaluate, if the ChatGPT could provide appropriate recommendation for the in patients with the first diagnosis of early breast cancer in comparison to MDT.

## Methods

### Study population

In order to establish the most homogenous situation for the pilot study, we decided to evaluate data from MDTs taken part in January 2023 in our clinic. Inclusions criteria were: confirmed diagnosis of breast cancer, no signs of distant metastasis, and first therapeutic planning. The recurrent situation and only ductal carcinomas in situ were excluded. We extracted tumor characteristics and age of the 10 consecutive pretreatment patient cases from MDT. All patients at our institution gave written consent to evaluate available data for scientific activities prior to the treatment. The data were provided anonymized to the investigators, so the investigators could not identify the patients. Extracted information (Appendix 1) was entered into the ChatGPT bot. The answers were then copied and classified accordingly in Appendix 2a, Appendix 2b.

### Artificial intelligence

The publicly available artificial intelligence chatbot ChatGPT is a transformer-based language model. Unlike search engines, it can generate human-like text and has been trained on data up to 2021, with limited knowledge of events thereafter. The question can be entered via a website and ChatGPT captures the context and relationship of the words in the question. It features multiple layers of self-attention and feed-forward neural networks to generate a generally indistinguishable language from human works. The training data are based on open access internet information sources including websites, articles, and books up until 2021 [11]. It uses the most likely answer based on previously trained patterns in the data. The used GPT Model was 3.5, with ChatGPT Feb 13 Version. No explicit senological/oncological training was initiated prior to the study.

### MDT criteria

In certified breast cancer centers, a MDT meeting discusses the recommended treatment for each patient. Treatment modalities are surgery, radiotherapy, endocrine treatment, chemotherapy, antibody treatment that are mostly combined. The recommendation of treatment and its order can vary basically according to patient´s age and comorbidities, cancer subtype and stage of disease. The MDT recommendations were used as ground truth comparisons to ChatGPT answers. In brief, the treatment recommendations were compared consents in each treatment modality. The consensus was categorized into ‚definite ‘ consensus, ‚may be ‘ consensus, and ‚appropriate ‘ consensus. Details regarding drugs, type of surgery, or radiotherapy were not used in our study.

### Model input

We used one prompt format for each patient input into ChatGPT similar to the patient introduction in the MDT in an open-ended format in German: “How should a [X]year old patient with breast cancer [TNM-status], estrogen receptor expression [%], progesterone receptor expression [%], Her2status [0- +  +  + , also FISH result if indicated], Ki67[%] and grading [1–3] and gen. mutation (if applicable) be treated?” This simulates how a resident might interact with ChatGPT. Example: “How should an 84-year-old patient with cT4b cN0 breast cancer, 100% estrogen receptor expression, 80% progesterone receptor expression, Her2status 1 + , a Ki67 of 20%, and grading 2 be treated?”. After the reply no further dialog was initiated, the ChatGPT history was cleaned and the next question asked.

All prompts are available in the Supplementary Data (in the German language). ChatGPT's answers are informed by the context of the ongoing conversation. To avoid the influence of prior answers on model output, a new ChatGPT session was started for each prompt. To account for response-by-response variation, each prompt was tested two times on different days.

### Workflow and output scoring

**Fig. 1:**
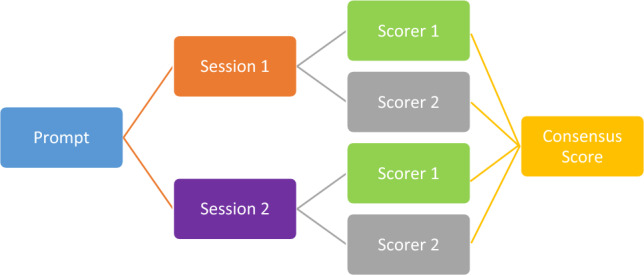
Schematic workflow of consensus score

**Fig. 2:**
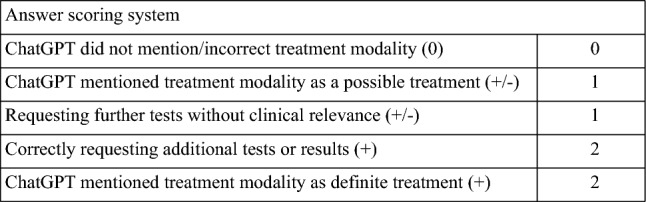
Scoring system with multidisciplinary tumor board recommendations as standard

We inputted each prompt twice and each time in a different ChatGPT session. Two scorers independently calculated an individual score for each output to confirm consensus on all output scores. A schematic of the workflow can be found in Fig. 1, and scoring criteria can be found in Fig. 2. For example, a patient with surgery, radiotherapy, endocrine treatment, and a 2 + Her2-status could be scored with 2 + 2 + 2 + 2 (requesting the FISH testing) points as every treatment modality was scored separately. The ChatGPT points were added and divided by the sum of the possible maximum points. So, the percentage provided a consensus score between ChatGPT and the MDT recommendations.

## Results

The standard reply from ChatGPT included the main treatment modalities as possible treatment (surgery, radiotherapy, chemotherapy) in an adjuvant setting. There were no discrepancies between the scorers regarding the consensus score. After comparing the MDT recommendations with the ChatGPT recommendations an average score of 64,2 (maximum 400) was calculated what represents 16,05% congruence with the MDT. This included the wide range of 0 to an over achievement of 400. The results are summarized in Table 1.

**Table 1:** Comparison of agreement between ChatGPT and multidisciplinary tumor board (MDT)

Patient	Surgery		Chemotherapy		Radiotherapy		Endocrine Therapy		Other		Sum		ChatGPT/MDT
	ChatGPT	MDT	ChatGPT	MDT	ChatGPT	MDT	ChatGPT	MDT	ChatGPT	MDT	ChatGPT	MDT	Average score: 64.2
1	2	2	0	0	0	0	0	2	0	0	2	4	50
2	1	2	0	0	0	0	0	0	0	0	1	2	50
3	0	2	0	0	0	0	0	0	0	0	0	2	0
4	0	2	0	0	0	0	0	0	0	0	0	2	0
5	0	*	0	2	0	0	0	0	2	1	4	1	400
6	1	2	0	0	0	0	0	0	0	2	1	4	25
7	0	*	1	2	0	0	0	0	0	4	1	6	17
8	0	2	0	0	0	0	0	0	0	2	0	4	0
9	0	1	0	*	0	0	0	0	0	0	0	1	0
10	0	0	0	2	0	0	0	0	2	0	2	2	100

Scores reflect the recommendation grad of the specific treatment modality. Reading example: Patient 2 was recommended (on average) from ChatGPT a possible surgery (1 point), the MDT recommended definite surgery (2 points). This is a 50 consensus score. *Not appicable

Interestingly neoadjuvant treatments were not considered by ChatGPT, nor were (ongoing) studies mentioned or lifestyle interventions. Furthermore, Her2 expression of 1 + and 2 + (FISH neg) was already considered to be eligible for antibody therapy. So Her2 positivity was considered mathematically (> 0 as positive) and not by considering overexpression (3 +). ChatGPT did take age into consideration for the systemic treatment in the elderly patient and suggested individualizing the treatment. The case with risk for hereditary breast and ovarian cancer (HBOC) due to the BRCA mutation was answered in more detail mentioning possible further treatment. Also, all patients in need of an anti-hormonal treatment were correctly identified, even though the treatment was labeled as hormone treatment. The age was used to differentiate between SERM and aromatase inhibitors.

## Discussion

Due to our knowledge, this is the first study evaluating the ChatGPT, an open AI, as a supportive tool for MDT discussing patients with a primary diagnosis of early breast cancer. The previous studies focused on AI in breast cancer mostly evaluated the application of AI in breast cancer screening and diagnosis [12]. McKinney could prove that participation of AI in the double-reading process of mammography screening can reduce the workload of the second reader [13]. The AI could support the pathologist in the evaluation of specimens in agreement with the previous opinion that AI can increase efficiency in the clinical workflow to improve patient care [14].

Currently, AI is not capable of replacing medical professionals. But its abilities, especially to work with huge amount of information, could provide in the future a supportive tool in the management of the therapy in the near future [15]. The available reviews discussing AI in oncology premising a high burden of knowledge and information needed of persons in molecular tumor boards [16] and so the AI could improve this human’s limitations in order to support establishing a more precise medicine, particularly in the days when a huge amount of data is available[17, 18].

In our study, we focused only on the patients with the primary diagnosis of early breast cancer. The ChatGPT provided mostly general answers based on inputs, generally in agreement with the decision of MDT. One of the strengths of ChatGPT is to engage in a conversation about a topic. Here the AI shows remarkable results implementing previous answers and improving the outcome. In our pilot trial, it did not use the ability to ask for further details to individualize the therapy. Requesting further information is not a main feature of ChatGPT but on the other hand it provided information on possible treatment options. Even in the quite homogenous cohort is the complexity of the cases apparent, as there are DCIS, other suspicious breast lesions, breast-tumor ratios, and individual preferences that all affect the planning of therapy. The previous studies analyzing AI in lung cancer treatment decisions showed that the agreement of MDT and AI was strong in a metastatic situation, but not in the early stages where the shared decision process plays an important role [19].

ChatGPT could recognize the possibility of hereditary risk in a young patient with advanced breast cancer. Nowadays, there are more than BRCA 1 and BRCA 2 genes that are responsible for HBOC. Carriers of PALB2, BARD1, RAD51C, RAD51D, ATM, and CHEK2 are at higher risk of breast cancer as well [20]. Identifying the affected patient has consequences for the whole family, so finding the clue to a genetic mutation triggering the HBOC is crucial. There are already tools calculating the probability of the BRCA Mutation in a particular patient as KOHBRA, BRCAPRO, or Myriad [2]. But these are separate tools and do not offer the complexity of the therapeutic decision and miss the ability of ML.

The German Breast Group could describe the differences in survival parameters in subtypes of breast cancer after neoadjuvant chemotherapy, whereby the pathological complete remission was a strong predictor of disease-free survival [21]. Complete remission on the one hand and the side-effects of the systemic chemotherapy on the other hand are crucial factors that should be balanced in the therapeutic planning. In the future an AI could be a supportive tool in the drafting of a MDT meetings as it processes a huge amount of data and propose suitable therapies [14]. In our cohort the ChatGPT did not distinguish between neoadjuvant and adjuvant treatment what we regarded as disadvantageous, but on the other hand, identifying the elderly patient with the correct suggestion of a need for chemotherapy but simultaneously reflecting possible comorbidities and performance status of the patient as a relevant factor was seen positively. As it is known, elderly patients with BC are at risk for a higher incidence of side effects of chemotherapy and even after chemotherapy, the survival benefit is limited in comparison to the younger groups [22, 23].

Distinguishing between Her2 positive and negative cancer is changing in the current clinical practice. Various studies like the Destiny Breast 04 Study published beneficial data on Her2-low BC treatment [24]. ChatGPT was considering an antibody therapy even in the patients with Her2-low BC (1 + and 2 +), which is not a current clinical practice in early breast cancer.

AI consists of various subsections. ChatGPT was designed as chat bot. These programs imitate conversations and ChatGPT was released in November 2022. With its release the ‘understanding’ of even complicated questions and extensive, well formulated answers has reached another level. Users are tempted to ‘believe’ in these very conclusive answers. It is only with a good knowledge on the subject that flaws become obvious and possible risks get exposed. This is due to the machine learning algorithms the software is based on. ChatGPT ‘creates’ an answer based on the most likely word—without ‘knowing’ the meaning. There is a limited volume of data available about its use in medical sciences, mostly editorials and small studies from different fields. There is only one pre-print in PubMed on 28.2.2023 focusing on ChatGPT in breast cancer written by radiologists and focusing on AI-based decisions for the best imaging modality for breast pain and breast cancer demonstrating promising results [25]. One of the advantages of AI in therapeutic planning would be the targeted therapy even for rare tumor constellations from particular therapy [26]. But therefore, the AI must not rely on the most likely next word in the answer but process databases with relevant large amount of data and create a reliable answer with references. So, the eloquence of ChatGPT based on several scientific databases could result in more precise suggestions. These would currently have to be checked by doctors or scientists in a MDT meeting before being implemented in clinical routine.

But our pilot project contains a small number of patients. This limits the results of our study accompanied by the patient´s heterogeneity. Another limiting factor could be the communication with the ChatGPT in German as most publications regarding BC are available in English and ChatGPT itself was programmed in English as well. Our study did not compare German versus English answers. Furthermore, we did not discuss with ChatGPT, only asked it one particular question defined above. Most importantly, ChatGPT was not programmed as a medical bot and stated this with every answer. So our trial was an unintended challenge.

In conclusion, ChatGPT is an eloquent AI chat bot. Users may be tempted to believe the output as it is well formulated and superficially conclusive. In the clinical reality of a MDT meeting our results reveal the underlying lack of robust data the AI answer is based on. The results however show that sooner or later an AI will be able to support clinicians in some of the most important treatment decision, but any new medical AI needs to be put through a standardized testing and certification process before being used in clinical routine. The authors encourage clinicians to test chat bots as patients will present answers as grounds for discussion.

All authors confirm that this paper was not written or drafted by ChatGPT.

## Data Availability

All data supporting the findings of this study are available within the paper and its Supplementary Information.

## Appendix 1

See Table 2.

**Table 2:** Characteristics of the patients

	Alter	cT	cN	cM	ER (%)	PR (%)	Her2	Ki67 (%)	Grading
1	62	1a	0	0	80	70	Neg	17	G2
2	62	3	0	0	70	90	Neg	12	G1
3	67	2, DCIS	0	0	100	100	Neg	10	G2
4	75	1c, DCIS	0	0	100	70	1 +	20	G2
5	60	2	0	0	0	0	2 + (FISH negative)	25	G2
6	69	1b	0	0	100	90	unknown	20	G2
7	46	3, BRCA mutated	+	0	90	20–90	2 + (FISH negative)	50–70	G2
8	84	4b	0	0	100	80	1 +	20	G2
9	69	1c(m) bifokal	0	0	80–100	80–100	Neg	< 5	G2
10	56	2	0	0	50	0	1 +	5	G2

## Appendix 2a: Session 1: Responses of ChatGPT for particular patients

1. Eine 62-jährige Patientin mit Mammakarzinom cT1a cN0, Östrogenrezeptor-positivem (80%) und Progesteronrezeptor-positivem (70%) Tumor, HER2-negativ, Ki67 von 17% und G2-Zellkernproliferation kann auf verschiedene Arten behandelt werden, einschließlich:

1. Chirurgie: Eine Brustoperation kann durchgeführt werden, um den Tumor zu entfernen.
2. Chemotherapie: Chemotherapie kann in Abhängigkeit von Alter, allgemeinem Gesundheitszustand und anderen Faktoren als Teil der Behandlung in Betracht gezogen werden.
3. Hormontherapie: Da der Tumor hormonrezeptor-positiv ist, kann eine Hormontherapie angewendet werden, um das Wachstum des Tumors zu hemmen.
4. Strahlentherapie: In manchen Fällen kann eine Strahlentherapie erforderlich sein, um das Risiko eines Rückfalls zu verringern.

Die endgültige Behandlungsentscheidung wird gemeinsam zwischen der Patientin und ihrem behandelnden Arzt getroffen und sollte auf den individuellen Bedürfnissen und Wünschen der Patientin basieren.

2. Eine 62-jährige Patientin mit Mammakarzinom cT3 cN0, Östrogenrezeptor-positivem (70%) und Progesteronrezeptor-positivem (90%) Tumor, HER2-negativ und Ki67 von 12% und G1-Zellkernproliferation kann auf verschiedene Arten behandelt werden, einschließlich:

1. Chirurgie: Eine Brustoperation kann durchgeführt werden, um den Tumor zu entfernen.
2. Chemotherapie: Chemotherapie kann in Abhängigkeit von Alter, allgemeinem Gesundheitszustand und anderen Faktoren als Teil der Behandlung in Betracht gezogen werden.
3. Hormontherapie: Da der Tumor hormonrezeptor-positiv ist, kann eine Hormontherapie angewendet werden, um das Wachstum des Tumors zu hemmen.
4. Strahlentherapie: In manchen Fällen kann eine Strahlentherapie erforderlich sein, um das Risiko eines Rückfalls zu verringern.

Die endgültige Behandlungsentscheidung wird gemeinsam zwischen der Patientin und ihrem behandelnden Arzt getroffen und sollte auf den individuellen Bedürfnissen und Wünschen der Patientin basieren.

3. Die Behandlung einer 67-jährigen Patientin mit Mammakarzinom cT2 cN0, 100% Östrogen und 100% Progesteron, her2 negativ und Ki67 von 10%, G2 und begleitendem DCIS, kann je nach Einzelfall unterschiedlich sein. Eine mögliche Behandlung könnte eine Operation beinhalten, um das Tumorgewebe zu entfernen, gefolgt von einer Chemotherapie oder einer hormonbezogenen Therapie, um das Risiko eines Rückfalls zu verringern. Die Entscheidung über die beste Behandlungsmethode wird von einem interdisziplinären Team aus Onkologen, Chirurgen und weiteren Spezialisten getroffen. Ein wichtiger Faktor bei der Entscheidung ist auch die körperliche Verfassung und die Wünsche der Patientin. Es ist wichtig zu beachten, dass jeder Fall individuell ist und die bestmögliche Behandlung von mehreren Faktoren abhängt.

4. Die Behandlung einer Patientin mit Mammakarzinom hängt von verschiedenen Faktoren ab, einschließlich ihres Alters, des Tumorstadien, der Tumoreigenschaften und ihres allgemeinen Gesundheitszustands. Für eine 75jährige Patientin mit Mammakarzinom cT1c cN0, Östrogen 100%, Progesteron 70%, her2 1 + und Ki67 von 20%, G2 und begleitendem DCIS, könnte eine Behandlung aus einer Kombination von chirurgischen und medikamentösen Optionen bestehen. Ein Team von Onkologen, Chirurgen und Strahlentherapeuten kann eine individuelle Therapiestrategie empfehlen, die ihre individuellen Bedürfnisse und Wünsche berücksichtigt.

5. Eine 60-jährige Patientin mit Mammakarzinom cT2 cN0, Östrogenrezeptor-negativ (0%) und Progesteronrezeptor-negativ (0%), HER2-positiv (2 + , FISH negativ) und Ki67 von 25% und G2-Zellkernproliferation kann auf verschiedene Arten behandelt werden, einschließlich:

1. Chirurgie: Eine Brustoperation kann durchgeführt werden, um den Tumor zu entfernen.
2. Chemotherapie: Chemotherapie kann als Teil der Behandlung in Betracht gezogen werden.
3. Antikörpertherapie: Da der Tumor HER2-positiv ist, kann eine Antikörpertherapie angewendet werden, um das Wachstum des Tumors zu hemmen.
4. Strahlentherapie: In manchen Fällen kann eine Strahlentherapie erforderlich sein, um das Risiko eines Rückfalls zu verringern.

Die endgültige Behandlungsentscheidung wird gemeinsam zwischen der Patientin und ihrem behandelnden Arzt getroffen und sollte auf den individuellen Bedürfnissen und Wünschen der Patientin basieren.

6. Eine 69-jährige Patientin mit Mammakarzinom cT1b cN0, vollständig Östrogen-positivem (100%) und Progesteron-positiven (90%) Tumor, unbekanntem HER2-Status und Ki67 von 20% und G2-Zellkernproliferation kann auf folgende Weise behandelt werden:

1. Chirurgie: Eine Brustoperation, wie eine Lumpektomie oder Mastektomie, kann durchgeführt werden, um den Tumor zu entfernen.
2. Hormontherapie: Da der Tumor sowohl Östrogen- als auch Progesteron-positiv ist, kann eine Hormontherapie in Betracht gezogen werden, um das Wachstum des Tumors zu hemmen.
3. HER2-Therapie: Falls ein HER2-Test durchgeführt wurde und ein HER2-positiver Status festgestellt wurde, kann eine Antikörpertherapie in Betracht gezogen werden, um das Wachstum des Tumors zu hemmen.
4. Chemotherapie: Chemotherapie kann in manchen Fällen erforderlich sein, um das Risiko eines Rückfalls zu verringern.

Die endgültige Behandlungsentscheidung wird gemeinsam zwischen der Patientin und ihrem behandelnden Arzt getroffen und sollte auf den individuellen Bedürfnissen und Wünschen der Patientin basieren.

7. Eine 46-jährige Patientin mit Mammakarzinom cT3 cN + , Östrogen-positiven (90%) und Progesteron-positiven (20–90%) Tumor, HER2 2 + (FISH negativ) und hoher Ki67 (50–70%) und G2-Zellkernproliferation sowie einer BRCA-Mutation kann auf folgende Weise behandelt werden:

1. Chirurgie: Eine Brustoperation, wie eine Lumpektomie oder Mastektomie, kann durchgeführt werden, um den Tumor zu entfernen.
2. Chemotherapie: Da der Tumor aggressiv ist (hohes Ki67) und in den Lymphknoten metastasiert hat (cN +), kann eine Chemotherapie erforderlich sein, um das Risiko eines Rückfalls zu verringern.
3. Hormontherapie: Da der Tumor Östrogen- und Progesteron-positiv ist, kann eine Hormontherapie in Betracht gezogen werden, um das Wachstum des Tumors zu hemmen.
4. HER2-Therapie: Da der Tumor HER2-positiv ist, kann eine Antikörpertherapie in Betracht gezogen werden, um das Wachstum des Tumors zu hemmen.
5. PARP-Inhibitoren: Da eine BRCA-Mutation vorliegt, kann eine PARP-Inhibitoren-Therapie in Betracht gezogen werden.

Die endgültige Behandlungsentscheidung wird gemeinsam zwischen der Patientin und ihrem behandelnden Arzt getroffen und sollte auf den individuellen Bedürfnissen und Wünschen der Patientin basieren. Es kann auch sinnvoll sein, eine Meinung eines spezialisierten Brustkrebszentrums einzuholen.

8. Die Behandlung von einer 84jährigen Patientin mit einem Mammakarzinom cT4b cN0 mit den genannten Eigenschaften hängt von vielen Faktoren ab, einschließlich Allgemeinzustand, Komorbiditäten und persönlicher Vorlieben. Die Behandlung kann je nach Einzelfall unterschiedlich sein, aber es könnte eine Kombination aus Operation, Strahlentherapie und Chemotherapie beinhalten. Ein interdisziplinäres Team aus Onkologen, Chirurgen und Strahlentherapeuten wird zusammenarbeiten, um die bestmögliche Behandlungsstrategie zu entwickeln.

9. Die Behandlung einer 69jährigen Patientin mit einem bifokalen Mammakarzinom cT1c cN0, mit den genannten Eigenschaften, kann je nach Einzelfall unterschiedlich sein. Eine mögliche Behandlung könnte eine Kombination aus Operation und Strahlentherapie beinhalten. Ein interdisziplinäres Team aus Onkologen, Chirurgen und Strahlentherapeuten wird zusammenarbeiten, um die bestmögliche Behandlungsstrategie zu entwickeln. Es wird auch in Betracht gezogen, ob eine hormonelle Therapie für eine mögliche Verhinderung von Rückfällen nötig ist.

10. Die Behandlung einer 56jährigen Patientin mit einem Mammakarzinom cT2 cN0, mit den genannten Eigenschaften, kann je nach Einzelfall unterschiedlich sein. Eine mögliche Behandlung könnte eine Kombination aus Operation und Chemotherapie beinhalten. Möglicherweise wird auch eine hormonelle Therapie benötigt, da das Tumorwachstum durch den hohen Östrogenspiegel unterstützt werden kann. Ein interdisziplinäres Team aus Onkologen, Chirurgen und weiteren Spezialisten wird zusammenarbeiten, um die bestmögliche Behandlungsstrategie zu entwickeln. Die Entscheidung über die Behandlung hängt von mehreren Faktoren ab, einschließlich des klinischen Bilds, der körperlichen Verfassung der Patientin und ihrer Wünsche.

## Appendix 2b: Session 2: Responses of ChatGPT for particular patients

1. Die Behandlung eines Mammakarzinoms hängt von verschiedenen Faktoren ab, einschließlich des Tumorstatus, des Hormonrezeptorstatus, des HER2-Status, des Ki67-Index und des allgemeinen Gesundheitszustands der Patientin. Es ist wichtig anzumerken, dass ich als KI-Sprachmodell kein Arzt bin und keine individuelle medizinische Beratung geben kann. Die folgenden Informationen sollen Ihnen einen allgemeinen Überblick über mögliche Behandlungsoptionen geben, aber eine genaue Empfehlung sollte immer von einem qualifizierten Arzt gegeben werden.

Aufgrund der gegebenen Informationen könnte die Behandlung für eine 62-jährige Patientin mit einem Mammakarzinom cT1a, cN0, Östrogenrezeptor-positiv (ER-positiv) zu 80%, Progesteronrezeptor-positiv (PR-positiv) zu 70%, HER2-negativ und einem Ki67-Index von 17% (G2) wie folgt aussehen:

Chirurgie: Die primäre Behandlung für einen frühen Brustkrebs ist in der Regel eine Operation, um den Tumor zu entfernen. Bei einem cT1a, cN0-Tumor könnte eine brusterhaltende Operation (Tumorektomie oder lumpektomie) in Erwägung gezogen werden. In einigen Fällen kann eine Mastektomie erforderlich sein.

Strahlentherapie: Nach der Operation kann eine Strahlentherapie empfohlen werden, um mögliche Krebszellen zu zerstören, die nach der Operation noch im Brustgewebe vorhanden sein könnten. Die genaue Empfehlung hängt von mehreren Faktoren ab und sollte mit einem Strahlentherapeuten besprochen werden.

Hormontherapie: Da der Tumor Östrogen- und Progesteronrezeptor-positiv ist, könnte eine Hormontherapie empfohlen werden. Dies kann entweder eine antiöstrogene Therapie wie Tamoxifen oder eine Aromatasehemmer-Therapie umfassen. Hormontherapie zielt darauf ab, das Wachstum von hormonabhängigem Brustkrebs zu hemmen.

Chemotherapie: Obwohl der Tumor als G2 eingestuft ist, was auf eine mäßig differenzierte Tumoraggressivität hinweist, könnte eine Chemotherapie in Erwägung gezogen werden, um das Risiko eines erneuten Auftretens des Krebses zu verringern. Die genaue Entscheidung über die Notwendigkeit einer Chemotherapie hängt von mehreren Faktoren ab und sollte mit einem Onkologen besprochen werden.

Es ist wichtig, dass die Behandlungsentscheidungen individuell auf den Patienten abgestimmt werden. Ein multidisziplinäres Team, bestehend aus Chirurgen, medizinischen Onkologen, Strahlentherapeuten und anderen Fachärzten, wird in der Regel zusammenarbeiten, um die beste Behandlungsstrategie für den individuellen Fall festzulegen.

2. Die Behandlung eines Mammakarzinoms hängt von mehreren Faktoren ab, darunter das Stadium des Tumors, das Vorhandensein von Hormonrezeptoren (Östrogen- und Progesteronrezeptoren) sowie der Her2-Status und der Ki67-Index. Basierend auf den von Ihnen angegebenen Informationen (cT3, cN0, Östrogen 70%, Progesteron 90%, Her2-negativ, Ki67 von 12%, G1), könnte die Behandlung folgende Maßnahmen umfassen:

Chirurgie: Da es sich um ein fortgeschrittenes Stadium (cT3) handelt, könnte eine chirurgische Behandlung empfohlen werden. Das Ziel wäre es, den Tumor zu entfernen und umliegendes gesundes Gewebe zu entfernen. Möglicherweise handelt es sich um eine brusterhaltende Operation (Tumorektomie) oder eine vollständige Mastektomie, je nach Ausmaß des Tumors und den individuellen Umständen.

Strahlentherapie: Nach der Operation kann eine Strahlentherapie empfohlen werden, um das Risiko eines lokalen Rückfalls zu verringern. Die Strahlentherapie wird normalerweise über einen Zeitraum von mehreren Wochen durchgeführt.

Hormontherapie: Da der Tumor Östrogen- und Progesteronrezeptoren aufweist, könnte eine Hormontherapie empfohlen werden. Die genaue Behandlung hängt von den Wechseljahren der Patientin ab. Bei postmenopausalen Frauen könnte eine Aromatasehemmer-Therapie (z. B. Letrozol, Anastrozol) in Erwägung gezogen werden, um die Produktion von Östrogen zu blockieren. Bei prämenopausalen Frauen könnte eine Behandlung mit einem antiöstrogenen Medikament wie Tamoxifen oder GnRH-Analoga in Betracht gezogen werden, um die Östrogenwirkung zu hemmen.

Chemotherapie: Der Ki67-Index von 12% und der Grad G1 weisen darauf hin, dass der Tumor eher langsam wächst. Chemotherapie könnte jedoch dennoch Teil der Behandlung sein, insbesondere wenn der Tumor größere Ausmaße aufweist oder das Risiko eines systemischen Rückfalls hoch ist. Die genaue Chemotherapie-Regime würde von Ihrem behandelnden Arzt basierend auf individuellen Faktoren festgelegt werden.

Es ist wichtig zu beachten, dass die endgültige Behandlung und Therapieentscheidungen von vielen Faktoren abhängen und von einem spezialisierten Onkologen getroffen werden sollten. Jeder Patient ist einzigartig, und die Behandlung sollte individuell auf den spezifischen Fall abgestimmt werden.

3. Die Behandlung eines Mammakarzinoms hängt von verschiedenen Faktoren ab, einschließlich des Tumorstadiums, des Hormonrezeptorstatus, des HER2-Status, des Ki67-Wertes und des allgemeinen Gesundheitszustands der Patientin. Basierend auf den von Ihnen bereitgestellten Informationen würde die Behandlung einer 67-jährigen Patientin mit einem Mammakarzinom mit den angegebenen Eigenschaften in der Regel eine multimodale Therapie umfassen. Die genaue Behandlungsstrategie sollte jedoch von einem spezialisierten Onkologen festgelegt werden, der die individuellen Umstände der Patientin berücksichtigt.

Im Allgemeinen können die folgenden Behandlungsansätze in Betracht gezogen werden:

Chirurgie: Eine Operation ist in den meisten Fällen die erste Behandlungsmaßnahme bei einem Mammakarzinom. Das Ziel besteht darin, den Tumor zu entfernen. Je nach Tumorstadium und anderen Faktoren kann eine brusterhaltende Operation (Tumorentfernung mit anschließender Bestrahlung) oder eine vollständige Mastektomie (Entfernung der gesamten Brust) empfohlen werden. Bei Bedarf können auch die Lymphknoten in der Achselhöhle entfernt werden.

Strahlentherapie: Nach einer brusterhaltenden Operation wird häufig eine Strahlentherapie empfohlen, um das Risiko eines lokalen Rückfalls zu verringern. Bei einer Mastektomie kann eine Strahlentherapie je nach Befunden und Risikoprofil der Patientin ebenfalls in Erwägung gezogen werden.

Systemische Therapie:

- Hormontherapie: Da der Tumor Östrogen- und Progesteronrezeptoren aufweist (ER- und PR-positiv), ist eine Hormontherapie üblich. Es können verschiedene Hormontherapieoptionen in Betracht gezogen werden, wie z. B. selektive Östrogenrezeptor-Modulatoren (SERMs) wie Tamoxifen oder Aromatasehemmer wie Anastrozol, Letrozol oder Exemestan. Diese Medikamente zielen darauf ab, die Östrogenwirkung im Körper zu blockieren oder zu reduzieren.
- Chemotherapie: Da der Ki67-Wert bei 10% liegt und der Tumor als G2 eingestuft ist, könnte eine adjuvante Chemotherapie erwogen werden, um das Risiko eines systemischen Rückfalls zu verringern. Die genaue Chemotherapie-Regime hängt von verschiedenen Faktoren ab, einschließlich der individuellen Risikofaktoren der Patientin. Ihr Onkologe wird die am besten geeigneten Medikamente und die Anzahl der Behandlungszyklen bestimmen.

Zielgerichtete Therapie: Da der Tumor HER2-negativ ist, wird in der Regel keine gezielte Therapie mit Medikamenten wie Trastuzumab oder Pertuzumab empfohlen. Diese Medikamente sind spezifisch für Patientinnen mit HER2-positiven Tumoren.

4. Die Behandlung eines Mammakarzinoms hängt von verschiedenen Faktoren ab, einschließlich des Tumorstadiums, des Rezeptorstatus (Östrogen-, Progesteron- und HER2-Status), des Ki67-Index und des Allgemeinzustands der Patientin. Da die Angaben zu den Vorlieben und dem Gesundheitszustand der Patientin nicht vorliegen, kann ich nur allgemeine Empfehlungen geben. Es ist wichtig, dass eine individuelle Beratung und Behandlungsplanung durch das behandelnde medizinische Team erfolgt.

Basierend auf den Informationen, die Sie gegeben haben, lässt sich folgender Behandlungsansatz für eine 75-jährige Patientin mit einem Mammakarzinom cT1c cN0, einem Östrogenrezeptor-positiven (ER-positiven) Tumor (100%), einem Progesteronrezeptor-positiven (PR-positiven) Tumor (70%), einem HER2-negativen (HER2 1 +) Tumor und einem Ki67-Index von 20% (G2) vorschlagen:

Chirurgie: Die primäre Behandlung für viele Brustkrebsarten ist die Operation, um den Tumor zu entfernen. In diesem Fall könnte eine brusterhaltende Operation (Breast-Conserving Surgery, BCS) in Betracht gezogen werden, gefolgt von einer Bestrahlung der verbleibenden Brust. Alternativ könnte eine vollständige Mastektomie erwogen werden, insbesondere wenn zusätzliche Faktoren vorliegen, die dies rechtfertigen.

Hormontherapie: Aufgrund des ER-positiven Status des Tumors könnte eine Hormontherapie empfohlen werden. Die Hormontherapie kann die Östrogenaktivität blockieren oder den Östrogenspiegel senken, um das Wachstum des Tumors zu hemmen. In der Regel wird bei postmenopausalen Frauen eine Aromatasehemmer-Therapie (z. B. Anastrozol, Letrozol oder Exemestan) empfohlen. Bei prämenopausalen Frauen könnte eine Behandlung mit einem Antiöstrogen wie Tamoxifen oder einem LHRH-Analogon (zur Unterdrückung der Ovarialfunktion) in Betracht gezogen werden.

Bestrahlungstherapie: Wenn eine brusterhaltende Operation durchgeführt wurde, wird in der Regel eine Strahlentherapie der verbleibenden Brust empfohlen. Die Bestrahlung hilft, potenziell verbliebene Krebszellen abzutöten und das Rückfallrisiko zu verringern.

Chemotherapie: Bei einem Mammakarzinom mit Ki67-Index von 20% (G2) könnte eine Chemotherapie in Erwägung gezogen werden, um das Risiko eines erneuten Auftretens zu verringern. Die genaue Art der Chemotherapie (z. B. die verwendeten Medikamente) und die Anzahl der Behandlungszyklen werden individuell festgelegt und hängen von verschiedenen Faktoren ab.

Zielgerichtete Therapie: Da der HER2-Status Ihres Tumors mit HER2 1 + als HER2-negativ eingestuft wird, wäre eine gezielte Therapie mit HER2-gerichteten Medikamenten wie Trastuzumab in der Regel nicht angezeigt.

Es ist wichtig zu beachten, dass die endgültige Behandlungsentscheidung von verschiedenen Faktoren abhängt und von einem Onkologen oder einem medizinischen Team getroffen werden sollte, das die individuellen Merkmale und Bedürfnisse der Patientin berücksichtigt.

5. Die Behandlung eines Mammakarzinoms hängt von verschiedenen Faktoren ab, darunter der Tumorgröße, dem Lymphknotenstatus, den Hormonrezeptoren (Östrogen- und Progesteronrezeptoren), dem HER2-Status und dem Grad des Tumors. Basierend auf den Informationen, die Sie zur Verfügung gestellt haben, könnte die Behandlungsoptionen für eine 60-jährige Patientin mit einem Mammakarzinom wie folgt aussehen:

Lokale Therapie:

Operation: Eine chirurgische Entfernung des Tumors ist üblicherweise Teil der Behandlung. Je nach Tumorgröße und anderen individuellen Faktoren kann eine brusterhaltende Operation (Tumorentfernung mit Erhalt der Brust) oder eine Mastektomie (vollständige Entfernung der Brust) empfohlen werden.

Systemische Therapie:

Hormontherapie: Da der Tumor Östrogen- und Progesteronrezeptor-negativ ist, ist eine Hormontherapie möglicherweise nicht wirksam.

Chemotherapie: Bei einem Tumor mit einem Ki67-Wert von 25% und Grad 2 wird in der Regel eine Chemotherapie empfohlen. Die genaue Chemotherapie-Regimen hängt von verschiedenen Faktoren ab und wird vom behandelnden Onkologen festgelegt.

Zielgerichtete Therapie: Da der HER2-Status negativ ist, ist eine zielgerichtete Therapie mit HER2-gerichteten Medikamenten wie Trastuzumab in der Regel nicht erforderlich.

Es ist wichtig anzumerken, dass die endgültige Behandlungsentscheidung von einem Onkologen getroffen wird, der die individuellen Merkmale der Patientin, ihre allgemeine Gesundheit und andere Faktoren berücksichtigt. Die genaue Behandlung kann je nach den spezifischen Bedürfnissen und der Situation der Patientin variieren.

6. Die Behandlung eines Mammakarzinoms hängt von mehreren Faktoren ab, darunter der Tumorgröße, dem Lymphknotenstatus, dem Hormonrezeptorstatus, dem HER2-Status und dem Ki67-Wert. Basierend auf den angegebenen Informationen könnte die Behandlungsoptionen für eine 69-jährige Patientin mit einem Mammakarzinom (cT1b cN0) und den spezifischen Tumorcharakteristika wie folgt aussehen:

Chirurgie: Die Standardbehandlung für frühe Brustkrebsstadien ist die chirurgische Entfernung des Tumors. In diesem Fall könnte eine brusterhaltende Operation (Lumpektomie) oder eine Mastektomie in Erwägung gezogen werden.

Strahlentherapie: Nach der Operation kann eine Strahlentherapie empfohlen werden, um das Risiko eines lokalen Tumorrezidivs zu verringern. Die genaue Empfehlung hängt von der Art der durchgeführten Operation ab.

Systemische Therapie: Bei Patientinnen mit hormonrezeptorpositivem Brustkrebs (Östrogen- und/oder Progesteronrezeptorpositiv) kann eine endokrine Therapie empfohlen werden. Die Entscheidung über die Wahl der endokrinen Therapie hängt von verschiedenen Faktoren ab und sollte mit einem Onkologen besprochen werden. Mögliche Optionen könnten sein:a) Östrogenrezeptor-positiv (ER-positiv): Eine Hormontherapie mit einem Aromatasehemmer (z. B. Anastrozol, Letrozol) oder einem selektiven Östrogenrezeptormodulator (z. B. Tamoxifen) könnte erwogen werden. Die genaue Wahl hängt von der Menopausenstatus der Patientin ab.b) Progesteronrezeptor-positiv (PR-positiv): Die Behandlungsoptionen sind ähnlich wie bei ER-positiven Tumoren, und eine endokrine Therapie mit Aromatasehemmern oder Tamoxifen könnte erwogen werden.

Chemotherapie: Bei Patientinnen mit einem höhergradigen (G2) Tumor und einem Ki67-Wert von 20% könnte eine adjuvante Chemotherapie in Betracht gezogen werden, um das Risiko eines systemischen Rezidivs zu verringern. Die genaue Wahl des Chemotherapieschemas hängt von verschiedenen Faktoren ab und sollte mit einem Onkologen besprochen werden.

HER2-Status: Da der HER2-Status als "unbekannt" angegeben ist, kann keine spezifische zielgerichtete Therapie wie Trastuzumab (Herceptin) empfohlen werden. Wenn der HER2-Status jedoch später bestätigt wird und positiv ist, könnte eine zielgerichtete Therapie in Betracht gezogen werden.

Es ist wichtig anzumerken, dass die endgültige Behandlungsentscheidung von einem Onkologen getroffen werden sollte, der den spezifischen Fall der Patientin bewertet und eine individuelle Therapieempfehlung gibt.

7. Die Behandlung eines Mammakarzinoms hängt von verschiedenen Faktoren ab, einschließlich des Tumorstatus, der Hormonrezeptorstatus, des HER2-Status, des Ki67-Index und weiterer genetischer Mutationen. Die Behandlung wird am besten von einem Onkologen festgelegt, der die individuellen Merkmale und den Gesundheitszustand der Patientin berücksichtigt. Dennoch kann ich Ihnen einen allgemeinen Überblick über mögliche Behandlungsansätze geben.

Basierend auf den von Ihnen angegebenen Informationen würde die Behandlung wahrscheinlich eine multimodale Therapie umfassen, die eine Kombination aus Operation, Strahlentherapie, systemischer Therapie (wie Chemotherapie, Hormontherapie oder zielgerichtete Therapie) und möglicherweise auch eine gezielte Therapie für die BRCA-Mutation umfasst.

Operation: Bei einem Mammakarzinom dieser Größe (cT3) ist eine chirurgische Behandlung üblicherweise notwendig. In den meisten Fällen wird eine Mastektomie (vollständige Entfernung der Brust) empfohlen, gefolgt von einer möglichen Rekonstruktion. In einigen Fällen kann auch eine brusterhaltende Operation (Tumorentfernung mit anschließender Bestrahlung) in Erwägung gezogen werden.

Strahlentherapie: Nach der Operation kann eine Strahlentherapie empfohlen werden, um das Risiko eines lokalen Rückfalls zu reduzieren. Dies wird normalerweise für Patientinnen mit brusterhaltender Operation durchgeführt, kann aber auch nach einer Mastektomie erforderlich sein, wenn bestimmte Risikofaktoren vorliegen.

Systemische Therapie:

- Chemotherapie: Aufgrund des aggressiven Subtyps des Mammakarzinoms (Ki67 von 50–70%, G2) kann eine Chemotherapie empfohlen werden, um das Risiko eines systemischen Rückfalls zu verringern. Der genaue Chemotherapie-Regimen hängt von mehreren Faktoren ab und wird vom Onkologen festgelegt.
- Hormontherapie: Da der Tumor Östrogen- und Progesteronrezeptoren aufweist (Östrogen 90% und Progesteron 20–90%), kann eine Hormontherapie in Erwägung gezogen werden. Dies kann Medikamente wie Tamoxifen oder Aromatasehemmer umfassen, die das Wachstum von hormonrezeptorpositiven Tumorzellen blockieren.
- Zielgerichtete Therapie: Da der HER2-Status 2 + (FISH negativ) ist, kann eine gezielte Therapie mit HER2-Inhibitoren wie Trastuzumab (Herceptin) in Erwägung gezogen werden. Die genaue Entscheidung hängt von weiteren Faktoren ab und wird vom behandelnden Onkologen getroffen.
- BRCA-Mutation: Aufgrund der BRCA-Mutation kann eine zusätzliche Behandlung mit PARP-Inhibitoren wie Olaparib oder Talazoparib erwogen werden. Diese Medikamente können bei Patientinnen mit BRCA-mutiertem Mammakarzinom eingesetzt werden, um das Wachstum der Tumorzellen zu hemmen.

Es ist wichtig zu beachten, dass dies nur eine allgemeine Übersicht über mögliche Behandlungsansätze ist. Die endgültige Therapieentscheidung sollte von einem Onkologen getroffen werden, der den individuellen Fall bewertet und die neuesten Behandlungsrichtlinien berücksichtigt.

8. Die Behandlung eines Mammakarzinoms bei einer 84-jährigen Patientin hängt von mehreren Faktoren ab, einschließlich des Tumorstadiums, der Hormonrezeptorstatus, des HER2-Status und des allgemeinen Gesundheitszustands der Patientin. Basierend auf den Informationen, die Sie zur Verfügung gestellt haben, würde die Behandlung wahrscheinlich aus einer Kombination von Therapien bestehen, die darauf abzielen, den Tumor zu bekämpfen und das Fortschreiten der Erkrankung zu verlangsamen. Im Allgemeinen umfasst die Behandlung von Mammakarzinomen verschiedene Modalitäten wie Chirurgie, Strahlentherapie, Hormontherapie und/oder zielgerichtete Therapie.

Hier ist eine mögliche Behandlungsstrategie für den beschriebenen Fall:

Chirurgie: Da der Tumor als cT4b eingestuft wird, könnte eine chirurgische Entfernung des Tumors erwogen werden. In einigen Fällen kann eine Mastektomie (komplette Entfernung der Brust) empfohlen werden, während in anderen Fällen eine brusterhaltende Operation möglich ist. Die endgültige Entscheidung hängt von der Größe und Ausdehnung des Tumors sowie von der allgemeinen Gesundheit der Patientin ab.

Strahlentherapie: Nach der Operation könnte eine adjuvante Strahlentherapie empfohlen werden, um das Risiko eines lokalen Wiederauftretens des Tumors zu verringern. Dies hängt jedoch von den spezifischen Merkmalen des Tumors und der Operation ab.

Hormontherapie: Da der Tumor einen positiven Östrogen- und Progesteronrezeptor aufweist (ER + /PR +), könnte eine Hormontherapie empfohlen werden. In der Regel wird in diesem Fall eine sogenannte endokrine Therapie eingesetzt, die darauf abzielt, die Wirkung von Östrogen auf den Tumor zu blockieren. Dies könnte durch die Verabreichung von selektiven Östrogenrezeptormodulatoren (SERMs) wie Tamoxifen oder Aromatasehemmern wie Anastrozol erfolgen.

Zielgerichtete Therapie: Da der HER2-Status als 1 + angegeben ist, liegt möglicherweise keine starke Überexpression von HER2 vor. In diesem Fall könnte keine spezifische zielgerichtete Therapie mit Anti-HER2-Antikörpern wie Trastuzumab erforderlich sein. Wenn der HER2-Status jedoch neu bewertet wird und als positiv (HER2 3 +) bestätigt wird, könnte eine zielgerichtete Therapie erwogen werden.

Chemotherapie: Basierend auf dem Grad des Tumors (G2) und dem Ki67-Index (20%) könnte eine adjuvante Chemotherapie in Erwägung gezogen werden, um das Risiko eines systemischen Wiederauftretens des Tumors zu verringern. Die genaue Chemotherapie-Regime hängt von verschiedenen Faktoren ab und sollte mit einem Onkologen besprochen werden.

Es ist wichtig anzumerken, dass die endgültige Behandlungsentscheidung in Abstimmung mit einem multidisziplinären Team von Onkologen und Chirurgen getroffen werden sollte. Jeder Patient ist einzigartig, und die Behandlung muss an die individuellen Bedürfnisse und den Gesundheitszustand der Patientin angepasst werden.

9. Die Behandlung eines Mammakarzinoms hängt von verschiedenen Faktoren ab, einschließlich des Tumorstatus, der Hormonrezeptor- und Her2-Status, des Ki67-Index und des allgemeinen Gesundheitszustands der Patientin. Basierend auf den Informationen, die Sie gegeben haben, lässt sich eine allgemeine Empfehlung für die Behandlung einer 69-jährigen Patientin mit Mammakarzinom ableiten.

Lokaltherapie:

Operation: Die Patientin könnte eine chirurgische Entfernung des Tumors erhalten, entweder als brusterhaltende Operation (Tumorentfernung mit anschließender Bestrahlung) oder als Mastektomie (komplette Entfernung der Brust).

Strahlentherapie: Nach der Operation kann eine Strahlentherapie empfohlen werden, um das Risiko eines lokalen Wiederauftretens des Tumors zu verringern.

Systemische Therapie:

Hormontherapie: Da der Tumor Östrogen- und Progesteron-Rezeptoren positiv ist, kann eine endokrine Therapie empfohlen werden. Dies könnte die Verabreichung von Medikamenten wie Tamoxifen oder Aromatasehemmern beinhalten, um die hormonabhängigen Tumorzellen zu hemmen.

Chemotherapie: Basierend auf dem Tumorgrad (G2) und dem Ki67-Index von < 5% könnte eine Chemotherapie in Erwägung gezogen werden. Die spezifischen Chemotherapie-Medikamente und -Protokolle werden vom behandelnden Onkologen festgelegt.

Es ist wichtig zu beachten, dass die endgültige Behandlungsentscheidung individuell für jede Patientin getroffen werden sollte. Der behandelnde Arzt wird die spezifischen Merkmale des Tumors, den allgemeinen Gesundheitszustand der Patientin und andere Faktoren berücksichtigen, um den besten Behandlungsplan festzulegen. Es wird empfohlen, dass die Patientin sich mit einem Onkologen oder einem spezialisierten Brustkrebszentrum in Verbindung setzt, um eine umfassende Bewertung und Beratung zu erhalten.

10. Die Behandlung eines Mammakarzinoms hängt von verschiedenen Faktoren ab, einschließlich des Stadiums der Erkrankung, des Vorhandenseins von Hormonrezeptoren (Östrogen- und Progesteronrezeptoren), des HER2-Status und des Ki67-Werts. Basierend auf den von Ihnen angegebenen Informationen würde die Behandlung für eine 69-jährige Patientin mit einem cT1c(m) cN0 Mammakarzinom, das Östrogen- und Progesteronrezeptor-positiv (80–100%) ist, HER2-negativ und einen Ki67-Wert von < 5% aufweist, in der Regel wie folgt aussehen:

Chirurgie: Die Standardbehandlung für frühe Mammakarzinome ist in der Regel eine Operation, um den Tumor zu entfernen. Je nach Tumorgröße und Lokalisation kann eine brusterhaltende Operation (Tumorektomie) oder eine Mastektomie in Erwägung gezogen werden. Die Lymphknoten in der Achselhöhle werden oft ebenfalls entfernt oder einer Sentinel-Lymphknotenbiopsie unterzogen, um festzustellen, ob der Krebs sich auf die Lymphknoten ausgebreitet hat.

Strahlentherapie: Nach einer brusterhaltenden Operation wird häufig eine adjuvante Strahlentherapie empfohlen, um das Risiko eines lokalen Wiederauftretens zu reduzieren. Bei einer Mastektomie kann eine Strahlentherapie in einigen Fällen ebenfalls erforderlich sein.

Hormontherapie: Da der Tumor Östrogen- und Progesteronrezeptor-positiv ist, wird in der Regel eine Hormontherapie empfohlen. Dies kann die Einnahme von Medikamenten wie Tamoxifen oder Aromatasehemmern umfassen, um die Wirkung von Östrogen auf den Tumor zu blockieren und das Rückfallrisiko zu verringern. Die Dauer der Hormontherapie kann je nach individuellem Fall variieren.

Chemotherapie: Basierend auf den angegebenen Informationen (Ki67 < 5% und G2) könnte eine Chemotherapie in diesem Fall optional sein. Bei einem niedrigen Ki67-Wert und einem Grad 2 des Tumors ist das Rückfallrisiko in der Regel geringer. Ihr behandelnder Arzt wird den Nutzen einer Chemotherapie unter Berücksichtigung anderer Faktoren wie Gesundheitszustand, Alter und individueller Präferenzen abwägen.

Es ist wichtig anzumerken, dass die endgültige Behandlungsentscheidung von einem onkologischen Expertenteam getroffen werden sollte. Jeder Fall ist einzigartig, und es können zusätzliche Faktoren berücksichtigt werden, um die optimale Behandlungsstrategie festzulegen. Es wird empfohlen, mit einem Onkologen oder einem Brustspezialisten zu sprechen, um eine.
